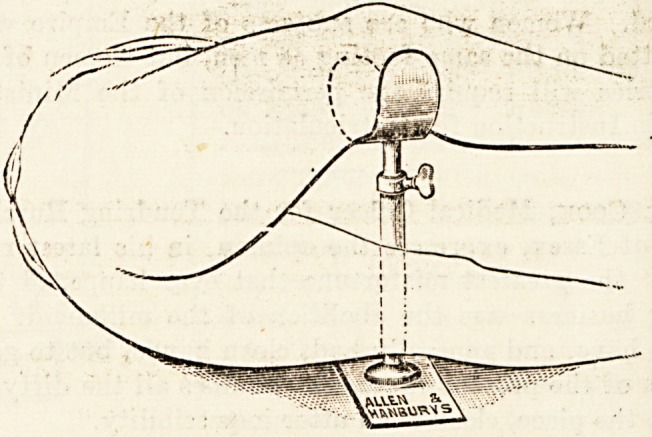# New Appliances and Things Medical

**Published:** 1908-08-29

**Authors:** 


### NEW APPLIANCES & THINGS MEDICAL.

### A NEW OBSTETRIC CRUTCH.

Dr. J. Reuell Atkinson, of Chadwell Heath, writes :— In obstetric operations, and especially when applying forceps with the patient lying on the left side, I have often been hampered by the right thigh and leg being in the way of my hands; and the nurse, to whom I usually give the duty of holding up the right leg, soon begins to find its weight tiresome. To obviate all this I have designed an obstetric crutch which has been made for me by Messrs. Allen and Hanburys (see woodcut). The crutch is made of aluminium and is light, strong, and cleanly.

By means of a screw the upper portion may be raised or lowered to any convenient height. I have had one in use in my practice for several months (it goes comfortably into my midwifery-bag), and both patients and nurses have expressed themselves much pleased with it. With the instrument in place the least touch upon the heel is sufficient to balance the leg, and occasionally I have managed without any such help from an attendant. Even in normal labours I have found the value of the crutch when the head is passing through the vulva.

## Figures and Tables

**Figure f1:**